# Microbial community structure and biogenic amines content variations in chilled chicken during storage

**DOI:** 10.1002/fsn3.3122

**Published:** 2023-01-02

**Authors:** Hong Min, Fengqiu An, Ting Wei, Song Wang, Pengfei Ma, Yong Dai

**Affiliations:** ^1^ NMPA Key Laboratory for Testing Technology of Pharmaceutical Microbiology Shaanxi Institute for Food and Drug Control Xi'an People's Republic of China; ^2^ School of Environmental and Chemical Engineering Xi'an Polytechnic University Xi'an People's Republic of China; ^3^ Shaanxi Institute for Food and Drug Control Xi'an People's Republic of China

**Keywords:** bacterial community structure and diversity, biogenic amine contents, chilled chicken, high‐throughput sequencing technique

## Abstract

The aim of this study was to investigate the sensory indicators, biogenic amine contents, and bacterial community structure and diversity of chilled chicken stored at 4°C under aerobic conditions. Bacterial diversity and dominant bacteria were analyzed using high‐throughput sequencing technique (HTS). The relationship between biogenic amine contents and microbial community structure was studied. The results showed that contents of putrescine and cadaverine increased significantly with storage time. Proteobacteria was absolutely dominant flora at the phylum level. The predominant spoilage bacteria found in chicken thighs were *Pseudomonas*, *Acinetobacter*, *Aeromonas*, *Shewanella*, and *Yersinia*, and the difference with chicken breasts was related to the presence of *Myroides* and absence of *Yersinia*. *Myroides*, *Yersinia*, and *Shewanella* were reported for the first time as an important contributor to the spoilage‐related microflora*.* Bacterial diversity and richness indices showed fluctuating and decreasing trend with storage time. The redundancy analysis showed that the relative abundance of *Pseudomonas*, *Yersinia*, and *Janthinobacterium* was positively related to the contents of putrescine, cadaverine, and tyramine, while *Shewanella* and *Aeromonas* showed positive relationship with putrescine content. Furthermore, positive relationship of *Myroides* and *Desulfovibrio* with the contents of cadaverine and tyramine was proposed for the first time. The key findings of this study can provide experimental data for food safety monitoring during refrigerated storage and preservation for poultry meat products.

## Graphical abstract

Proteobacteria was absolutely dominant flora at the phylum level. The predominant spoilage bacteria found in chicken thighs were *Pseudomonas*, *Acinetobacter*, *Aeromonas*, *Shewanella*, and *Yersinia*, and the difference with chicken breasts was related to the presence of *Myroides* and absence of *Yersinia*. *Myroides*, *Yersinia*, and *Shewanella* were reported for the first time as an important contributor to the spoilage‐related microflora. The redundancy analysis showed that the relative abundance of *Pseudomonas*, *Yersinia*, and *Janthinobacterium* was positively related to the contents of putrescine, cadaverine, and tyramine, while *Shewanella* and *Aeromonas* showed positive relationship with putrescine.

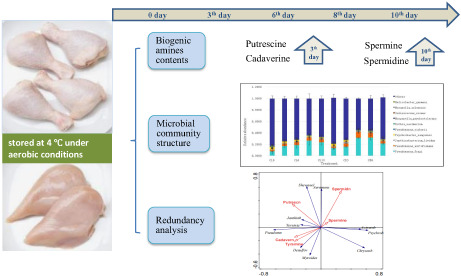

Chicken meat is characterized by high protein content and low fat and cholesterol levels, which are highly desired by the consumers. However, this kind of meat is vulnerable to the microbial contamination during production, storage, transportation, and marketing (Kim et al., 2019). Microorganisms with souring effect can proliferate in chicken meat by changing its pH and volatile nitrogen contents such as tyramine, putrescine, cadaverine, and other biogenic amines (Ivanov et al., 2015; Balamatsia et al., 2006; Balamatsia et al., 2007), thereby affecting the meat quality and food safety of chicken products.

Chicken could be contaminated by a variety of microorganisms during storage process. At present, the research on microbial community structure and diversity during the storage of meat products was conducted by various approaches. Among them were microbial culture methods combined with 16S rDNA sequencing technology (Zhang et al., 2015; Olsson et al., 2003), microbial culture methods combined with identity of fatty acid profiles of the isolates (Hinton et al., 2004), and denaturing gradient gel electrophoresis (DGGE; Liang et al., 2012; Jiang et al., 2011; Dias et al., 2013; Bekaert et al., 2015; Bassey et al., 2021). However, to the extent of our knowledge, HTS has not been studied yet or the microbial community structure and diversity during the storage of meat products. The traditional methods of microbial culture in which use of selective medium for specific microorganism growth is laborious have many limitations (Mohammed et al., 2020). This could be due to complex microbial composition in meat, and only 1% of the total microbial stains found in nature can be cultured on selective medium (Giraffa & Neviani, 2001). DGGE technology could not accurately reflect the microbial community structure and diversity during the storage of meat products.

HTS can sequence hundreds of thousands to millions of DNA molecules in one time, and targeted resequencing is a great application in HTS, so its read is shorter than previous sequencing technique. HTS is characterized by perfect quantitative function, ability to detect DNA species, and can test the abundance of species in samples (Qi et al., 2021). Furthermore, HTS can highlight predominant microorganisms from the phylum, class, order, family, genus, and species levels, thus reflecting deeper analysis of microbial community structure and diversity (Chen et al., 2021). HTS has been used generally to study the communities of the environment and human on the ground (Chen et al., 2020), but only a few studies have used this technique to analyze microbial communities of chilled poultry meat.

This work deals with the issues of chilled poultry meat, such as short shelf life, low meat quality, and food safety by investigating the sensory indicators, biogenic amine contents, and bacterial community structure and diversity. Microbial diversity and dominance of specific strains were analyzed by HTS. The relationship between biogenic amine contents and microbial community structure was examined. Some deterioration phenomena like mucous, moldy, foaming, and strong rotten sour odor were investigated in chilled chicken. The microbial mechanism that affects chilled chicken putrefaction was explained.

## 1 MATERIALS AND METHODS

### 1.1 Materials

Chilled chicken thighs and breasts were obtained from Furun Co., Ltd. slaughtering and processing unit. About 10 kg chicken thighs and 15 kg chicken breasts were divided into 10 sterile plastic sampling bags and labeled as CL0, CL3, CL6, CL8, and CL10 for chicken legs and CB0, CB3, CB6, CB8, and CB10 for chicken breasts. The samples were transported immediately to the laboratory in enclosed polystyrene type of packaging unit having ice blocks (4°C) under aerobic environment. The CL0 and CB0 were chosen to explore the microbial flora, structure, and diversity at the same day, while other samples were stored at 4°C under aerobic environment and analyzed for the microbial diversity after 3rd, 6th, 8th, and 10th days, respectively. To minimize the error each treatment was repeated 3 times.

### 1.2 Sensory analysis and evaluation

Sensory assessment of all the collected samples was performed by seven experts who have rich experience in chicken meat evaluation. Color, texture, appearance, and odor of chicken breast and thigh samples were evaluated. Sensory score criteria of chilled chicken during storage was shown in Table 1. A three‐class evaluation scheme was used: Class 1, fresh meat; Class 2, secondary fresh meat; and Class 3, spoiled meat.

**TABLE 1:** Sensory score criteria of chilled chicken during storage

Color	Appearance	Exudation	Odor	Score
Red	Moist, no mucus	Clear	No odor	4
Dark red	Mucous	A little opaque	Faint rotten sour odor	3
A little browning	Mucous, moldy	Opaque	Rotten sour odor	2
Some browning	Mucous, moldy, foaming	–	Strong rotten sour odor	1

### 1.3 Biogenic amines contents

The biogenic amines contents (tyramine, histamine, putrescine, cadaverine, spermine, spermidine, tryptamine, phenylethylamine) were determined according to SN/T 2209‐2008 and GB/T 5009.208‐2016. Samples were repeated 3 times for every date of storage. The biogenic amines were extracted with 0.4 M HClO_4_ solution and derived with dansyl chloride. The derivatization of amines results in dansyl derivates formation, which were detected by reverse‐phase HPLC of ultraviolet absorption at 254 nm wave length. The derivatized process was carried out by incubation for 40 min in 2 M NaOH solution and then buffered with NaHCO_3_, finally the sample was dissolved in acetonitrile. The derivatized samples were filtered (0.22 μm) and applied on a column (Cogent column HPS C18, ZORBAX SB‐Aq, 250 × 4.6 mm, 5 μm; Cogent) of a chromatographic system (pump and auto sampler Lab Alliance), degasser, UV/VIS‐DAD detector (λ = 254 nm), and column thermostat (Agilent Technologies, Agilent). The conditions for separation and detection of BA were described in GB5009.208‐2016; 1.7‐heptanediamine (Sigma‐Aldrich) was used as an internal standard.

### 1.4 Microbial community structure and diversity

#### 1.4.1 DNA extraction

The DNA of cold stored chicken thigh and breast samples were extracted according to the manufacturer's protocol of E.Z.N.A bacterial DNA extraction kit (Omega Bio‐Tek, Inc.). Samples were extracted 3 times for every date of storage. The quantity and quality of the extracted DNA were examined using a NanoDrop® ND‐2000c UV‐Vis spectrophotometer (NanoDrop Technologies).

#### 1.4.2 PCR amplification and Illumina sequencing

The V_4_ hypervariable regions of the 16S rRNA gene were amplified from each sample using the PCR primers 515F and 806R. The PCRs were conducted in 50 μl mixture, containing 20 μl Premix Ex Taq (TaKaRa), 0.4 μl of each primer (10 μM), 4 μl of fivefold diluted template DNA (1–10 ng), and 25.2 μl sterilized water (Qiagen). Thermal cycling conditions were as follows: an initial denaturation of 3 min at 94°C, six touchdown cycles of 45 s at 94°C, 60 s from 65 to 58°C, 70 s at 72°C, followed by 22 cycles of 45 s at 94°C, 60 s at 58°C, 60 s at 72°C with a final elongation of 72°C for 10 min. The PCR products were purified using a AxyPrep DNA Gel Extraction Kit (Axygen Biosciences). Thirty samples were sequenced on the Miseq platform (Illumina) at Novogene.

### 1.5 Bioinformatic analysis

After sequencing, initial DNA fragments were congregated by using FLASH (Magoc & Salzberg, 2011). Quantitative insights into microbial ecology (QIIME; Caporaso et al., 2010) and UPARSE pipeline (Edgar, 2013) software packages were employed to analyze each sequence reads assigned to every sample by their barcode. At first, QIIME quality filters were used to filter the sequence reads and then clustered into operational taxonomic units (OTUs) at a 97% identity threshold using UPARSE pipeline. The commissary sequence of every OTU was chosen and designated to taxonomic data by utilizing RDP classifier (Wang et al., 2007).

The microbial diversity was analyzed using QIIME V1.7.0 and displayed with R software (Version 2.15.3; Caporaso et al., 2010). Alpha diversity analysis included witnessed species, Ace and Chao1 estimators, Simpson and Shannon diversity indices, and Good's estimate of coverage. The term “Simpson's diversity index” reflected to Simpson's index (D), Simpson's index of diversity (1–D), and Simpson's reciprocal index (1/D). This article used the Simpson's index of diversity (1–D), which denotes the likelihood that two individuals haphazardly chosen from a sample will belong to various species.

### 1.6 Statistical analysis

Whole experiment was repeated 3 times for every date of storage. Based on the results of all sample species annotations, the top 10 phyla and the top 10 genera were examined (the relative abundance over 1%). A principal coordinate analysis (PCoA) was performed on the weighted UniFrac distance and unweighted pair group method with arithmetic mean (UPGMA) and shown by QIIME software (version 1.7.0).

In addition, correlations between key bacterial community composition and biogenic amine contents were studied by *t*‐value examination obtained from redundancy analysis (RDA) and conducted using Canoco 4.5 (Biometrics). The analyses data were acquired by Microsoft EXCEL (2016) spread sheets.

One‐way anova using SPSS 19.0 (SPSS Inc.) were used to analyze the contents of biogenic amines (tyramine, histamine, putrescine, cadaverine, spermine, spermidine, tryptamine, phenylethylamine), bacterial diversity indices (Shannon index, Simpson index), and richness indices (Chao 1 index, ACE index). Significance of difference was estimated by least significant differences (LSD) at *p* < .05.

## 2 RESULTS

### 2.1 Sensory analysis and evaluation of chilled chicken during storage

Sensory analysis and evaluation of chilled chicken during storage at 4°C under aerobic conditions was shown in Table 2. It can be seen that chilled chicken stored for 0 day was fresh meat, at third day was secondary fresh meat, and after sixth day of storage it was spoiled.

**TABLE 2:** Sensory analysis and evaluation of chilled chicken during storage

Samples	Color	Appearance	Exudation	Odor	Total score	Evaluation results
CL0	Red	Moist, no mucus	Clear	No odor	16	Fresh meat
CL3	Dark red	Moist, no mucus	A little opaque	No odor	14	Secondary fresh meat
CL6	A little browning	Mucous	Opaque	Faint rotten sour odor	10	Spoiled meat
CL8	Some browning	Mucous	Opaque	Rotten sour odor	8	Spoiled meat
CL10	Some browning	Mucous, moldy, foaming	Opaque	Strong rotten sour odor	5	Spoiled meat
CB0	Red	Moist, no mucus	Clear	No odor	16	Fresh meat
CB3	Dark red	Moist, no mucus	A little opaque	No odor	14	Secondary fresh meat
CB6	Dark red	Moist, no mucus	Opaque	Faint rotten sour odor	12	Spoiled meat
CB8	Some browning	Mucous, moldy	Opaque	Rotten sour odor	7	Spoiled meat
CB10	Some browning	Mucous, moldy, foaming	Opaque	Strong rotten sour odor	5	Spoiled meat

### 2.2 Biogenic amine contents of chilled chicken during storage

The types and contents of biogenic amines detected in chicken thighs and breasts during storage at 4°C under aerobic conditions are described in Table 3. The biogenic amines, tryptamine, phenylethylamine, and histamine, were not detected in chicken thighs and breasts during the whole storage period. The tyramine was not yielded at the initial stage of storage, however, the tyramine contents increased significantly in chicken thighs after sixth day and in breasts after eighth day of storage. Furthermore, the concentrations of putrescine and cadaverine also increased significantly in chicken thighs after sixth day and in breasts after eighth day of storage. As compared with the freshly slaughtered chicken, putrescine concentrations in chicken thighs after 3rd, 6th, 8th, and 10th days of storage increased by 629.06%, 823.58%, 1625.27%, and 2432.67%, respectively. On the other hand, cadaverine concentrations after 6th, 8th, and 10th days increased by 86.05%, 1223.85%, and 1361.20%, respectively. The putrescine concentrations in breasts of freshly slaughtered chicken increased by 21.78%, 24.68%, 364.38%, and 1034.12% after the 3rd, 6th, 8th, and 10th days of cold storage, respectively, while cadaverine concentrations increased by 73.75%, 53.35%, 434.79%, and 1302.81%, respectively. The concentration of spermine and spermidine did not significantly change during storage until 8th day, but increased significantly after 10th day.

**TABLE 3:** Change of biogenic amine contents of chilled chicken during storage

Samples	Tryptamine (mg/kg)	Phenylethylamine (mg/kg)	Putrescine (mg/kg)	Cadaverine (mg/kg)	Histamine (mg/kg)	Tyramine (mg/kg)	Spermidine (mg/kg)	Spermine (mg/kg)
CL0	0.00	0.00	12.06 ± 0.31 a	8.69 ± 0.84 a	0.00	0.00 a	17.53 ± 0.47 c	41.75 ± 2.94 b
CL3	0.00	0.00	12.00 ± 0.32 a	63.38 ± 3.31 b	0.00	0.00 a	13.37 ± 0.06 a	27.47 ± 4.12 a
CL6	0.00	0.00	22.44 ± 0.57 b	80.29 ± 3.20 b	0.00	12.16 ± 0.09 b	15.14 ± 0.48 b	36.47 ± 0.10 b
CL8	0.00	0.00	159.70 ± 8.33 c	149.98 ± 17.87 c	0.00	55.54 ± 0.33 c	13.55 ± 0.85 a	41.20 ± 2.61 b
CL10	0.00	0.00	176.27 ± 0.47 d	220.17 ± 3.95 d	0.00	92.88 ± 2.14 d	24.18 ± 0.20 d	64.95 ± 4.23 c
CB0	0.00	0.00	9.32 ± 0.02 a	33.03 ± 3.56 a	0.00	0.00 a	11.47 ± 0.18ab	32.07 ± 1.14 a
CB3	0.00	0.00	11.35 ± 0.12 a	57.39 ± 0.69 a	0.00	0.00 a	10.48 ± 0.48 a	26.74 ± 4.73 a
CB6	0.00	0.00	11.62 ± 0.36 a	50.65 ± 20.60 a	0.00	0.00 a	11.67 ± 0.67ab	27.24 ± 0.63 a
CB8	0.00	0.00	43.28 ± 1.26 b	176.64 ± 0.71 b	0.00	83.59 ± 1.16 b	10.36 ± 0.25 a	32.26 ± 0.33 a
CB10	0.00	0.00	105.70 ± 16.97 c	463.21 ± 24.63 c	0.00	192.34 ± 2.07 c	17.68 ± 0.26 c	74.95 ± 3.43 b

*Note*: Values are mean ± SD of three replicates. Biogenic amine contents within a column with no letters in common are significantly different (*p* < .05).

### 2.3 Microbial community structure of chilled chicken during storage

#### 2.3.1 Microbial community structure at the phylum level

**FIGURE 1:**
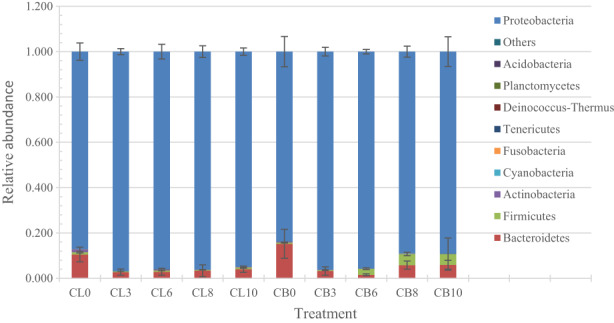
Taxonomic classification of bacterial reads retrieved from different samples at phylum level. Others represent the relative abundance of all other phyla outside the 10 phyla.

The soil bacterial communities at the phylum level of chicken thighs and breasts during storage at 4°C under aerobic conditions existed remarkable difference as shown in Figure 1. The dominant phyla of chicken thighs were Proteobacteria, Bacteroidetes, Firmicutes, and Actinobacteria, and the average relative abundance and standard deviation were 0.8720 ± 0.0381, 0.1049 ± 0.0326, 0.0104 ± 0.0034, and 0.0116 ± 0.0063, respectively, while the average relative abundance and standard deviation after 10 days were 0.9510 ± 0.0160, 0.0404 ± 0.0141, 0.0061 ± 0.0019, and 0.0008 ± 0.0002, respectively. The dominant phyla of chicken breasts were Proteobacteria, Bacteroidetes, Firmicutes, and Actinobacteria. the average relative abundance and standard deviation were 0.8376 ± 0.0672, 0.1523 ± 0.0640, 0.0056 ± 0.0020, and 0.0039 ± 0.0020, respectively, while the average relative abundance and standard deviation after 10 days were 0.8522 ± 0.0628, 0.0568 ± 0.0187, 0.0453 ± 0.0681, and 0.0008 ± 0.0001, respectively. Proteobacteria was the absolutely dominant flora at the phylum level.

### 2.4 Microbial community structure at the genus level

**FIGURE 2:**
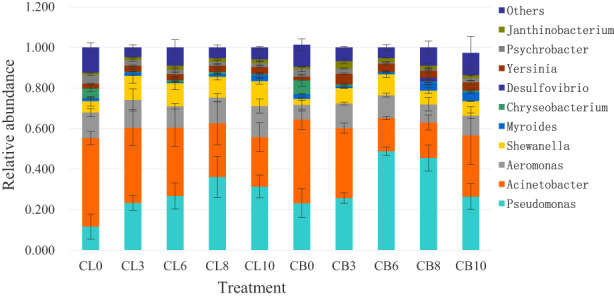
Taxonomic classification of bacterial reads retrieved from different samples at genus level. Others represent the relative abundance of all other phyla outside the 10 genera.

Figure 2 represents the bacterial communities at the genera level of chicken thighs and breasts during storage at 4°C under aerobic environment. The dominant genera of freshly slaughtered chicken thighs were *Acinetobacter*, *Aeromonas*, *Pseudomonas*, *Shewanella*, *Chryseobacterium*, *Psychrobacter*, and *Yersinia*, and the average relative abundance and standard deviation were 0.4373 ± 0.0324, 0.1254 ± 0.0195, 0.1165 ± 0.0618, 0.0559 ± 0.0242, 0.0484 ± 0.0163, 0.0447 ± 0.0061, and 0.0235 ± 0.0006, respectively, while the average relative abundance and standard deviation after 10 days were 0.2439 ± 0.0717, 0.1529 ± 0.0347, 0.3188 ± 0.0559, 0.1237 ± 0.0150, 0.0042 ± 0.0008, 0.0176 ± 0.0085, and 0.0353 ± 0.0053, respectively. As the storage duration prolongs, the average relative abundance of *Pseudomonas*, *Shewanella*, and *Yersinia* increased significantly, while *Acinetobacter*, *Chryseobacterium*, and *Psychrobacter* decreased significantly. To summarize, the overwhelming deterioration microorganisms of chicken thighs were *Pseudomonas*, *Acinetobacter*, *Aeromonas*, *Shewanella*, and *Yersinia*.

The dominant genera found in freshly slaughtered chicken breasts were *Acinetobacter*, *Pseudomonas*, *Aeromonas*, *Chryseobacterium*, *Psychrobacter*, *Shewanella*, and *Myroides*, and their average relative abundance and standard deviation were 0.4124 ± 0.0501, 0.2322 ± 0.0722, 0.0727 ± 0.0315, 0.0681 ± 0.0307, 0.0367 ± 0.0133, 0.0293 ± 0.0070, and 0.0235 ± 0.0025, respectively. The average relative abundance and standard deviation after 10 days were 0.3014 ± 0.1425, 0.2646 ± 0.0635, 0.0981 ± 0.0129, 0.0078 ± 0.0066, 0.0204 ± 0.0125, 0.0715 ± 0.0016, and 0.0443 ± 0.0072, respectively. With the prolonging storage time, the average relative abundance of *Pseudomonas*, *Shewanella*, and *Myroides* increased significantly, while *Chryseobacterium* decreased significantly. To sum up, the predominant spoilage bacteria of chicken breasts were *Acinetobacter*, *Pseudomonas*, *Aeromonas*, *Myroides*, and *Shewanella*.

### 2.5 Microbial community structure at the species level

**FIGURE 3:**
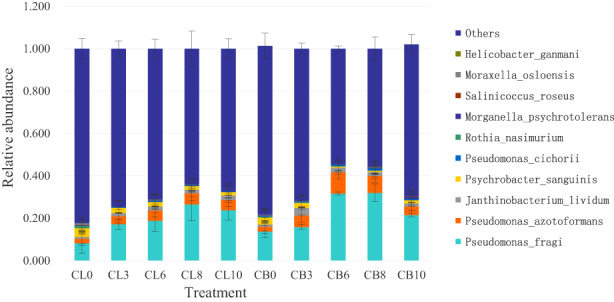
Taxonomic classification of bacterial reads retrieved from different samples at species level. Others represent the relative abundance of all other phyla outside the 10 species.

The average relative abundance of *Pseudomonas fragi*, *Psychrobacter sanguinis*, *Pseudomonas azotoformans*, *Rothia nasimurium*, *Janthinobacterium lividum*, *Moraxella osloensis*, *Morganella psychrotolerans*, and *Pseudomonas cichorii* in freshly slaughtered chicken thighs were 0.0819 ± 0.0473, 0.0414 ± 0.0057, 0.0207 ± 0.0107, 0.0097 ± 0.0058, 0.0093 ± 0.0030, 0.0088 ± 0.0012, 0.0024 ± 0.0009, and 0.0019 ± 0.0004, respectively, while the average relative abundance of dominant species in chicken thighs after 10 days of storage were 0.2377 ± 0.0459, 0.0159 ± 0.0079, 0.0492 ± 0.0083, 0.0003 ± 0.0001, 0.0200 ± 0.0081, 0.0009 ± 0.0002, 0.0094 ± 0.0028, and 0.0035 ± 0.0007, respectively (Figure 3). Similarly, dominant species of freshly slaughtered chicken breasts were *P. fragi*, *Psy. sanguinis*, *P. azotoformans*, *J. lividum*, *M. osloensis*, *P. cichorii*, and *R. nasimurium*, and their average relative abundance were 0.1371 ± 0.0285, 0.0335 ± 0.0117, 0.0219 ± 0.0060, 0.0118 ± 0.0029, 0.0039 ± 0.0014, 0.0036 ± 0.0009, and 0.0026 ± 0.0015, respectively, while the average relative abundance of dominant microbial species stored for 10 days were 0.2146 ± 0.0088, 0.0153 ± 0.0150, 0.0402 ± 0.0121, 0.0149 ± 0.0024, 0.0010 ± 0.0007, 0.0064 ± 0.0029, and 0.0002 ± 0.0001, respectively.

With the prolonging storage time, the average relative abundance of *P. fragi*, *P. azotoformans*, *Morg. psychrotolerans*, and *P. cichorii* in chicken thighs increased significantly, while *P. fragi* and *P. azotoformans* in chicken breasts increased significantly. To sum up, *P. fragi*, *P. azotoforman*, *Psy. sanguinis*, and *J. lividum* were predominant spoilage bacteria of chicken thighs and breasts.

### 2.6 Microbial community diversity of chilled chicken during storage

#### 2.6.1 Microbial diversity index

The OTUs richness, alpha diversity indices, and sample coverage of bacteria are shown in Table 4. Approximately 100% coverage values were seen in all samples. Bacterial diversity indices (Shannon index, Simpson index) and richness indices (Chao 1 index, ACE index) showed fluctuating and decreasing trend with storage time. Shannon diversity indices of chicken thighs decreased significantly after 3rd, 8th, and 10th days of storage. After 3rd, 6th, 8th, and 10th days of storage, the Chao1 richness indices of chicken thighs decreased by 9.55%, 18.84%, 23.65%, and 19.86%, while ACE richness indices decreased by 10.18%, 19.32%, 25.84%, and 23.05%, respectively.

**TABLE 4:** Microbial diversity index of chilled chicken during storage at 4°C under aerobic conditions

Treatments	Shannon index	Simpson index	Chao1 index	ACE index	Coverage rate (%)
CL0	4.417 ± 0.027 a	0.922 ± 0.003 a	345.652 ± 33.413 a	360.987 ± 45.282a	99.83
CL3	3.895 ± 0.084 b	0.896 ± 0.007 a	312.629 ± 135.204a	324.222 ± 93.112a	99.80
CL6	4.290 ± 0.208 a	0.909 ± 0.014 a	280.525 ± 58.275a	291.249 ± 52.245a	99.70
CL8	3.839 ± 0.270 b	0.877 ± 0.036 a	263.905 ± 36.251a	267.710 ± 40.890a	99.87
CL10	3.887 ± 0.080 b	0.886 ± 0.014 a	276.998 ± 44.429a	277.765 ± 13.812a	99.87
CB0	4.381 ± 0.103 a	0.925 ± 0.003 a	300.861 ± 55.099 a	312.134 ± 60.192 a	99.83
CB3	4.142 ± 0.079a	0.920 ± 0.003 a	221.768 ± 32.182a	238.077 ± 41.027a	99.90
CB6	3.761 ± 0.144 a	0.858 ± 0.006 b	293.013 ± 91.167a	305.536 ± 107.815a	99.73
CB8	4.106 ± 0.353 a	0.865 ± 0.027 b	264.345 ± 54.497a	273.349 ± 51.653a	99.83
CB10	4.362 ± 0.445 a	0.912 ± 0.031 a	238.150 ± 17.721a	250.354 ± 9.671a	99.80

*Note*: Values are mean ± standard deviation of three replicates. Bacterial diversity indices (Shannon index, Simpson index) and richness indices (Chao 1 index, ACE index) within a column with no letters in common are significantly different (*p* < .05).

Furthermore, chicken breasts stored at 4°C for 3rd, 6th, 8th, and 10th days also show decreasing trend of Shannon and Simpson diversity indices, Chao 1 and ACE richness indices. Simpson diversity indices of chicken breasts decreased significantly after 3rd and 8th days of storage. After 3rd, 6th, 8th, and 10th days of storage, the Chao1 richness indices of chicken breasts decreased by 26.29%, 2.61%, 12.14%, and 20.84%, while ACE richness indices decreased by 23.73%, 2.11%, 12.43%, and 19.79%, respectively.

### 2.7 PCoA and UPGMA clustering

**FIGURE 4:**
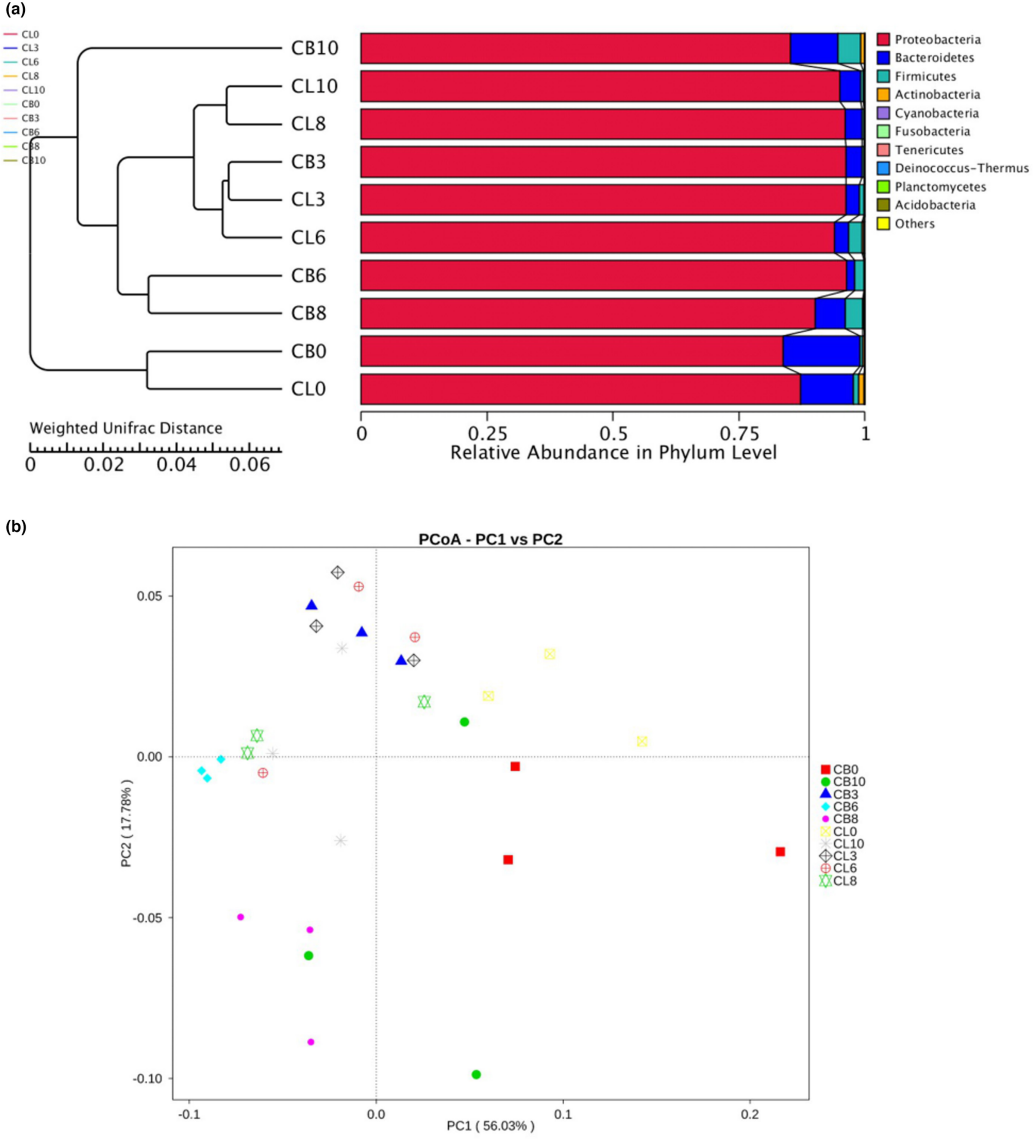
(a) Unweighted pair group method with arithmetic mean (UPGMA) clustering were conducted on the unweighted UniFrac distance. (b) Principal coordinate analysis (PCoA) plot based on the weighted UniFrac metric.

Unweighted pair group method with arithmetic mean (UPGMA) cluster analysis of microbial communities showed in Figure 4a. It can be seen that the samples CB3, CL3, and CL6 form cluster, while CL8 and CL10 assemble together, CB6 and CB8 were close to each other, CL0 were close to CB0. This grouping was further confirmed by PCoA and results were shown in Figure 4b. The variance contribution of first principal components (PC1) and second principal components (PC2) of the microbial communities were 56.03% and 17.78%, respectively, and the cumulative variance contribution was 73.81%. Cluster analysis and principal coordinate analysis showed that the storage time significantly affected the microbial community structure of chicken meat.

### 2.8 Correlations among microbial community structure and biogenic amine contents

**FIGURE 5:**
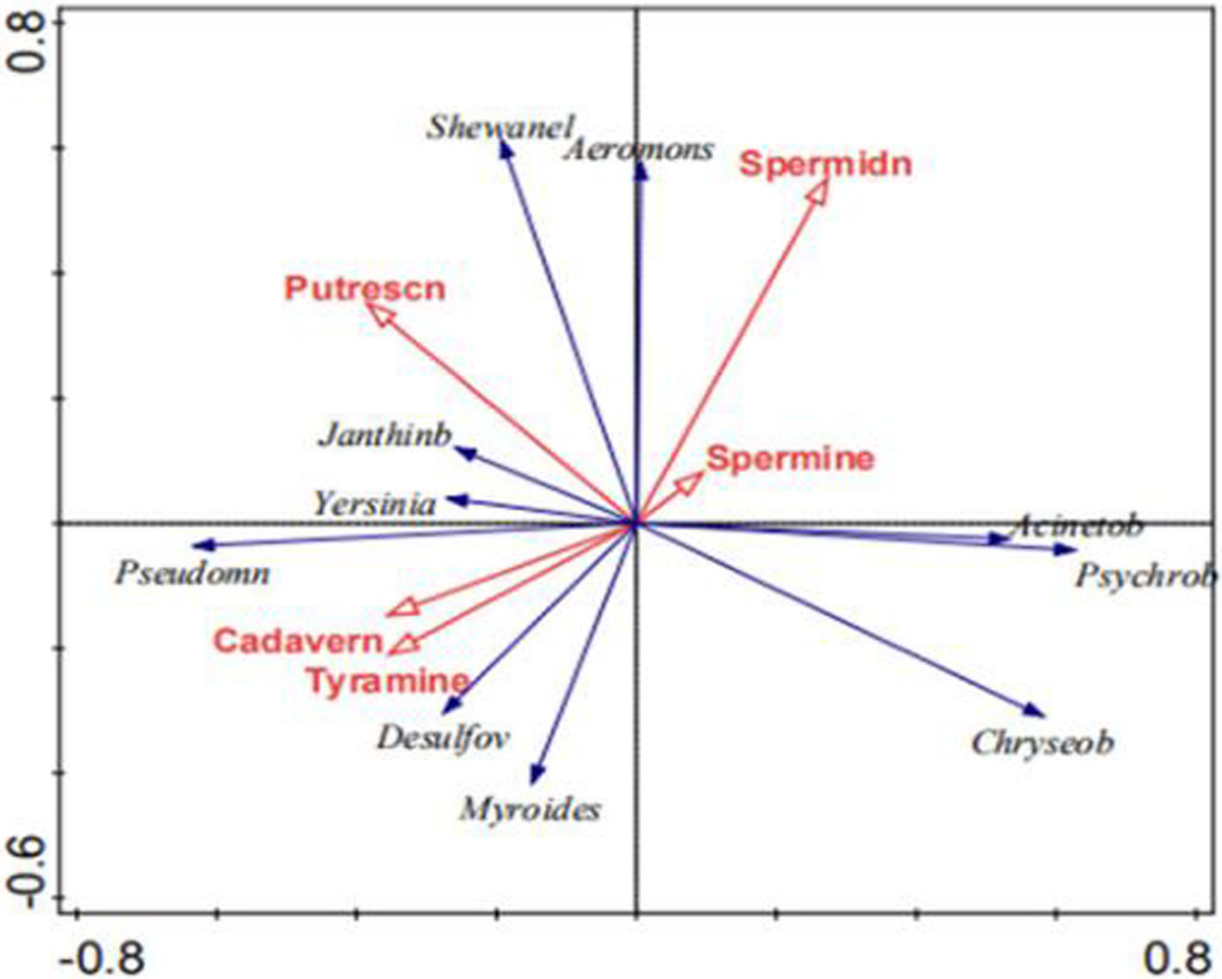
Redundancy analysis ordination of the five chicken variables and abundance of 10 dominant genus communities from chicken thighs and breasts stored at 4°C under aerobic conditions for 0, 3, 6, 8, and 10 days. RDA 1 and 2 explained 31.6% and 3.1% of the variance, respectively.

Figure 5 represents the RDA of five variables and abundance data from 10 dominant genera in the communities. The first axis of ordination explained 31.6% of the total variance (*p* = .001; Monte Carlo permutation test with 1000 permutations) and second axis of ordination explained 3.1% of the total variance. The combination of variables explained 36.8% of the total variance of genus abundances. The RDA showed that the relative abundance of *Pseudomonas*, *Yersinia*, and *Janthinobacterium* were positively related to the contents of putrescine, cadaverine, and tyramine, while those of *Shewanella* and *Aeromonas* showed positive relationship with putrescine. Moreover, the analysis proposed that relative abundances of *Myroides and Desulfovibrio* showed positive relationship with contents of cadaverine and tyramine.

## 3 DISCUSSION

### 3.1 Biogenic amine contents of chilled chicken during storage

Biogenic amines were considered as important indicators of meat freshness and spoilage, they have low molecular weight and consist of nitrogen‐containing aliphatic, aromatic, or heterocyclic compounds and normally produced due to the decarboxylation of amino acids (Baixas‐Nogueras et al., 2003). In brief, amino acids were decarboxylated either by endogenous amino acid decarboxylase that existed naturally in animal or plant cells or by exogenous enzymes generated by decarboxylase‐positive microorganisms under favorable environment and conditions (Fausto et al., 2016). The spoilage microorganisms often secrete amino decarboxylase and lead to accumulation of biogenic amines in meat and meat products. The biogenic amines histamine, putrescine, tyramine, tryptamine, β‐phenylethylamine, and cadaverine may be formed during storage of meat or during processing of these products (Balamatsia et al., 2006).

Naturally higher levels of spermine were found in fresh chicken (Silva & Glória, 2002; Patsias et al., 2006), which were agreed with the results of this study. Many studies showed that the contents of tyramine, cadaverine, putrescine, and histamine in refrigerated chicken meat were increasing as storage duration prolongs (Ivanov et al., 2015; Balamatsia et al., 2007; Triki et al., 2018), while spermine and spermidine were decreasing during storage (Balamatsia et al., 2006). In this study, the tyramine contents increased significantly in chicken thighs after sixth day and in breasts after eighth day of storage. Furthermore, the concentrations of putrescine and cadaverine also increased significantly in chicken thighs after sixth day and in breasts after eighth day of storage. While spermine and spermidine contents did not change significantly while stored at 4°C for 0, 3, 6 and 8 days, but increased significantly after 10th day of storage, indicating that the microbial population of arginine metabolizing increased sharply after 10th day of storage. Cadaverine and putrescine have a stale and abhorrent taste (Curiel et al., 2011), therefore refrigerated chicken at 4°C from 6th day produced faint rotten sour odor.

### 3.2 Microbial community structure and diversity

Meat putrefaction is an ecological phenomenon and normally caused by prevailing of a particular microbial strains called specific spoilage organism (SSO). The prevailing of specific microbial association on fresh meat mainly relies upon initial microflora and storage conditions (Zhang et al., 2015; Hovda et al., 2007).

The initially found microflora in meat products were closely related to the environment of manufacturing enterprise, equipment, personnel, operation, and kinds of meat products (Rudy et al., 2020). The microbial community structure and dominant microorganisms of meat products were different under different storage conditions such as *Aeromonas* (Zhang et al., 2015), *Carnobacterium* (Zhang et al., 2015), *Lactococcus* and *Lactobacillus* (Jääskeläinen et al., 2016), and Enterobacteriaceae (Kokeala & Bjrkroth, 1997) grow and reproduce under vacuum‐packaged cryopreserved conditions. Similarly, under air‐packaged cryopreserved conditions, the dominant specific spoilage organism were *Pseudomonas* (Hinton et al., 2004; Liang et al., 2012; De Filippis et al., 2013; Jiang et al., 2011; Koutsoumanis et al., 2006; Bekaert et al., 2015; Hovda et al., 2007; Zhang et al., 2015), *Brochothrix* (Liang et al., 2012), *Brochothrix thermosphacta* (De Filippis et al., 2013; Koutsoumanis et al., 2006), *Acinetobacter* (Hinton et al., 2004), *Aeromanas* (Hinton et al., 2004), *Psychobacter* (Bekaert et al., 2015), *Moraxlla* (Li et al., 2008), *Photobacterium* (Hovda et al., 2007), *Shewanella putrefaciens* (Hovda et al., 2007), *Rahnella* (Ercolini et al., 2006), *Rahnella aquatilis* (Ercolini et al., 2006), *Carnobacterium* (Liang et al., 2012), and *Carnobacterium divergens* (Ercolini et al., 2006). *Pseudomonas* (Hovda et al., 2007; Ercolini et al., 2006), *Leuconostoc* (Jääskeläinen et al., 2016), and *Lactobacillus sakei* (Ercolini et al., 2006) were the dominant bacteria in low temperature storage with high oxygen concentration.

In this study, the average relative abundance and standard deviation of *Pseudomonas*, *Acinetobacter*, *Aeromonas*, and *Shewanella* in chicken thighs after 10 days were 0.3138 ± 0.0559, 0.2439 ± 0.0717, 0.1529 ± 0.0347, and 0.1237 ± 0.0150, respectively, and *Acinetobacter*, *Pseudomonas*, *Aeromonas*, *Shewanella*, and *Myroides* in chicken breasts were 0.3014 ± 0.1425, 0.2646 ± 0.0635, 0.0981 ± 0.0129, 0.0715 ± 0.0016, and 0.0443 ± 0.0072, respectively. This study showed that *Pseudomonas*, *Acinetobacter*, *Aeromonas*, *Shewanella*, and *Yersinia* were specific spoilage organism (OOS) found in the chilled chicken thighs during storage at 4°C under aerobic conditions, while the difference in chicken breasts were related with the presence of *Myroides* and absence of *Yersinia*. *Pseudomonas* (Hinton et al., 2004; Liang et al., 2012), *Acinetobacter* (Hinton et al., 2004), *Carnobacterium* (Liang et al., 2012), *Brochothrix* (Liang et al., 2012), and *Aeromonas* (Hinton et al., 2004) were main spoilage microflora found in chilled chicken stored at 4°C under aerobic conditions in previous report. This research had some difference from previous studies, and the reason may be closely related to the different initial microbial community structure in freshly slaughtered chicken produced by different enterprises (Liang et al., 2012). *Pseudomonas* sp., an obligate aerobe, grew optimally at 30°C, but can proliferate at temperatures as low as 4°C (Fonseca et al., 2011), so it was an absolutely dominant spoilage bacteria in chilled chicken during storage at 4°C under aerobic conditions in this study, and *P. fragi*, *P. azotoformans*, and *P. cichorii* were the dominant spoilage bacterial species. *Pseudomonas* found in meat products has developed resistance to a variety of antibiotics (Lerma et al., 2012; Lerma et al., 2014), so its growth and reproduction should be tightly controlled.

In this study, we found that *Myroides*, *Yersinia*, and *Shewanella* were important contributors to the spoilage‐related microflora of chilled chicken during storage at 4°C under aerobic conditions. *Myroides* were aerobic, gram‐negative, rod‐shaped opportunistic pathogen (Schröttner et al., 2014) and widely distributed in nature (Cho et al., 2011; Ktari et al., 2012; Ramasamy et al., 2013; Zhang et al., 2014). *Yersinia* were facultative anaerobic, gram‐negative, rod‐shaped bacterium of the Enterobacteriaceae family (Fàbrega & Vila, 2012). *Yersinia enterocolitica* and *Yersinia pseudotuberculosis* were thought to be significant foodborne pathogens and were responsible of yersiniosis in humans and animals (Fukushima et al., 2011). *Shewanella* were gram‐negative motile bacillus, facultative anaerobe (Janda, 2014), and were potential pathogen of seawater and freshwater fish (Altun et al., 2014), as well as the main cause of spoiling of chilled fish (Gram & Dalgaard, 2002), while *S. putrefaciens* was the dominant spoilage bacteria for drumsticks stored in low‐temperature vacuum packaging (Sun et al., 2011).

As a psychrophilic organism, *Yersinia* was able to grow at 4°C (Grahek‐Ogden et al., 2007). *Myroides* and *Shewanella* also have ability to thrive at low temperatures (Hau & Gralnick, 2007), therefore the relative abundance of *Yersinia*, *Myroides*, and *Shewanella* increased with storage time in this study, revealing that *Myroides*, *Yersinia*, and *Shewanella* were potentially pathogenic to poultry safety. Therefore, cold chain transport and storage could offer a potential food safety hazard and must be controlled tightly.

Alpha diversity refers to the analysis of species richness and diversity of a single sample within a particular region or ecosystem, including Chao1, ACE, and Shannon and Simpson indices (Patrick et al., 2009). In this study, OTU coverage rates of all samples were above 99.70%, indicating that sequencing data were sufficient and biological information in the samples could be reflected completely, which can be used for the analysis of the microbial community diversity (Li et al., 2016). Chao1 and ACE indices indicated the species richness in samples, while Shannon and Simpson indices reflected species diversity (Wen et al., 2016). In case of same species richness in the community, the greater the species evenness, the greater the species diversity (Caporaso et al., 2010). In this study, Chao1, ACE, and Shannon and Simpson indices showed fluctuating and decreasing trend with storage time, revealing that bacterial diversity and richness decreased of chilled chicken during storage at 4°C under aerobic condition.

### 3.3 Correlations among microbial community structure and biogenic amine contents

According to reported literatures, the formation of biogenic amines in stored meat products were closely related to the decarboxylase‐secreted spoilage microbial flora. A number of bacteria produce biogenic amines, for example, strains of the family Enterobacteriaceae (Durlu‐Ö et al. 2001; Curiel et al. 2011), *Pseudomonas* (Rezaei et al. 2007), *Aeromonas* (Bunkova et al. 2010), *Lactobacilli* (Edwards et al. 1987), *Lactobacillus* (Arena et al. 2001; Edwards et al. 1987), *S. putrefaciens* (Wang et al. 2014), *Aeromonas veronii* (Wang et al. 2014), *Brochothrix thermosphacta* (Nowak et al. 2011), and also lactic acid producing bacteria (Bunkova et al. 2009; Lorencova et al. 2012).

The relative abundances of *Pseudomonas* and *Yersinia* were positively related to the contents of putrescine, cadaverine, and tyramine in this study. The relationship among the growth of *Pseudomonas* and cadaverine and putrescine was inseparable by regression analysis (Liu et al. 2012). Rezae et al. (2007) also found that the best linear correlations were for *Pseudomonas* spp. originated from farmed rainbow trout stored in ice and putrescine (*r* = .98), with cadaverine (*r* = .82). *Yersinia rohdei* and *Yersinia kritensenii* were identified as putrescine and cadaverine producer strains (Curiel et al. 2011).

Bunkova et al. (2010) reported that the 11 bacterial strains isolated from poultry skin classified into *Aeromonas* genus produced putrescine, and five of them also formed cadaverine. *Aeromonas verpnii* revealed a strong ability to produce putrescine and cadaverine. Wang et al. (2014) believed that *S. putrefaciens* showed significantly higher abilities to produce putrescine than those from other genera. This would support our hypothesis that the relative abundance of *Aeromonas* and *Shewanella* were positively related to putrescine contents in this study.

In this work, we proposed that the relative abundances of *Myroides* and *Desulfovibrio* showed positive relationship with cadaverine and tyramine contents.

## 4 CONCLUSIONS

In this work, we found the tyramine was not yielded at the initial stage of storage, however, the tyramine contents increased significantly in chicken thighs after 6th day and in breasts after 8th day of storage. Furthermore, the concentrations of putrescine and cadaverine also increased significantly in chicken thighs after 6th day and in breasts after 8th day of storage. The concentration of spermine and spermidine did not significantly change during storage until 8th day, but increased significantly after 10th day.

Proteobacteria was the absolutely dominant flora of chilled chicken during storage at the phylum level. The predominant spoilage bacteria found in chicken thighs were *Pseudomonas*, *Acinetobacter*, *Aeromonas*, *Shewanella*, and *Yersinia* at the genus level, and the difference with chicken breasts were related with the presence of *Myroides* and absence of *Yersinia*. *Myroides*, *Yersinia*, and *Shewanella* were reported for the first time as an important contributor to the spoilage‐related microflora. *Pseudomonas fragi*, *P. azotoforman*, *Psy. sanguinis*, and *J. lividum* were predominant spoilage bacteria of chicken thighs and breasts at the species level.

Bacterial diversity indices and richness indices showed fluctuating and decreasing trend with storage time. The RDA showed that the relative abundance of *Pseudomonas*, *Yersinia*, and *Janthinobacterium* was positively related to the contents of putrescine, cadaverine, and tyramine, while *Shewanella* and *Aeromonas* exhibit positive relationship with putrescine. Furthermore, positive relationship of *Myroides* and *Desulfovibrio* with the contents of cadaverine and tyramine was proposed.
